# Regular smoking of male ancestors in adolescence and fat mass in young adult grandchildren and great-grandchildren

**DOI:** 10.12688/wellcomeopenres.17950.1

**Published:** 2022-07-08

**Authors:** Steven Gregory, Matthew Suderman, Kate Northstone, Marcus Pembrey, Sarah Watkins, Yasmin Iles-Caven, Jean Golding

**Affiliations:** 1 Bristol Medical School (Population Health Sciences), University of Bristol, Bristol, Bristol, BS8 2BN, UK

**Keywords:** ALSPAC, cigarette smoking, adolescence, grandparents, fat mass, lean mass, intergenerational effects, great-grandparents

## Abstract

**Background:** Previous studies using the Avon Longitudinal Study of Parents and Children (ALSPAC) have shown that if men commenced smoking prior to the onset of puberty their sons, their granddaughters and great-granddaughters were more likely to have excess fat (but not lean) mass during childhood, adolescence and early adulthood. In this study we assess associations between ancestral smoking during adolescence (ages 11–16 years) with fat and lean mass of subsequent generations at two ages.

**Methods: **We analysed data on exposures of grandparents and great-grandparents collected by ALSPAC. The outcomes were the fat masses of their grandchildren and great-grandchildren measured at ages 17 and 24. Measures of lean mass were used as controls. Adjustment was made for 8–10 demographic factors using multiple regression.

**Results: **We found associations between adolescent smoking of the *paternal* grandfathers and the adjusted fat mass of their grandchildren, but no associations with the grandchildren’s lean mass. Grandchildren at age 17 had an average excess fat mass of +1.65 [95% CI +0.04, +3.26] Kg, and at age 24 an average excess of +1.55 [95% CI -0.27, +3.38] Kg. Adolescent smoking by the *maternal* grandfather showed similar, but weaker, associations: at 17 an average excess fat mass of +1.02 Kg [95% CI -0.20, +2.25] Kg, and at 24 an average excess of +1.28 [95% CI -0.11, +2.66] Kg. There were no pronounced differences between the sexes of the children. For the great-grandparents there were few convincing results, although numbers were small.

**Conclusions:** We have shown associations between grandfathers’ smoking in adolescence and increased fat (but not lean) mass in their children. Confirmation of these associations is required, either in a further data set or by demonstrating the presence of supportive biomarkers.

## Introduction

 The major spur towards the initiation of recent studies examining associations between human ancestral exposures and their descendants was a detailed analysis comparing the survival of individuals born in Sweden on the edge of the Arctic Circle between 1880 and 1915. Three cohorts of individuals were identified, based on year of birth occurring in the village of Őverkalix. Exposures to harvest glut and/or famine during the childhood of grandparents was identified and details linked to their grandchildren’s health indices. Detailed analyses highlighted the following effects on the grandchild: (i) there were strong relationships which were sex-specific, both in regard to the sex of the exposed grandparent and of the affected grandchild’s sex; and (ii) the exposure effects were specific to particular ages of exposure – the most susceptible being the years prior to puberty ^1^.

 This study prompted a number of projects assessing associations between exposures during the pre-pubertal period and health and development of the grandchildren. For example, van den Berg and Pinger studied the children and grandchildren of individuals who were exposed to the Berlin famine at ages 8–12 years. They demonstrated that those whose *mothers* had been exposed during these ages had worse health outcomes, particularly if they were male. Subsequently in the next generation, those granddaughters had higher (better) mental health scores if their *maternal* grandmothers were exposed to the famine pre-puberty, and those grandsons whose *paternal* grandfathers had pre-pubertal famine exposure had higher mental health scores ^2^.

 Among major cohort studies, information on environmental exposures during the childhood of parents has been collected occasionally, and rarely in the grandparents. The Avon Longitudinal Study of Parents and Children (ALSPAC) was one pre-birth cohort which collected information on the ages at which the parents of the index children had started smoking regularly. These data were used to ascertain whether index children whose parents had a history of starting to smoke regularly pre-puberty were likely to have differing growth patterns than those who started smoking later. We showed that if fathers had commenced regular smoking prior to the age of 11, their sons (but not their daughters) were more at risk of an increased body mass index (BMI), largely associated with excess fat mass at ages 13, 15 and 17 ^3^. Subsequently, a detailed study of antecedents associated with fat mass at age 24 indicated that the association remained with *paternal* smoking <11, and increased in size on adjustment ^4^. However, this study also showed an adjusted association between fat mass of the offspring and *maternal* onset of smoking during adolescence (i.e. at ages 11–16).

 We have subsequently determined whether the pre-pubertal ages at commencement of regular smoking of grandparents and/or great-grandparents was also associated with fat mass of the grandchildren and great-grandchildren. We compared the fat mass measurements of the different generations according to whether their ancestors had started smoking pre-puberty with those who started smoking in adolescence (11–16) ^5^. We hypothesised that any effects would differ according to the sex of both the ancestral smoker, and that of the grandchild and great-grandchild. In order to provide a comparison with the results for fat mass, we analysed the results for lean mass, and specifically looked at the outcomes of early onset smoking of the great-grandparents, grandparents and parents on the body composition of the index offspring in late adolescence and early adulthood. The results showed that granddaughters, but not grandsons, whose paternal grandfather commenced smoking pre-puberty (<13) were significantly fatter than those whose paternal grandfathers commenced smoking between the ages of 13 and 16. There were similar associations with the great-granddaughters (but not great-grandsons) of fathers of maternal grandfathers who had started pre-puberty ^5^. The analyses did not compare grandchildren and great-grandchildren of those ancestors who smoked during adolescence with those who did not. This is the aim of the present study.

Here we hypothesise: (i) that there are likely to be differences between the subsequent generations of children who started smoking before age 17 and those who either never smoked or who started smoking after age 16; (ii) that these are likely to vary with sex of the grandchildren and/or great-grandchildren, as well as with (iii) the mode of inheritance (i.e. whether down the maternal or paternal line). 

## Methods

### The ALSPAC population

 ALSPAC was designed to assess the ways in which aspects of the environment and genes of individuals interact to result in disadvantages or benefits to health and development. Pregnant women who were residents in a predefined area of Avon with an expected date of delivery between April 1991 and December 1992 inclusive were recruited. Eligible women were contacted as early in pregnancy as feasible. Initial numbers enrolled were 14,541 pregnancies (and at least one questionnaire had been returned or at least one attendance by mid-September 1999 at a “Children in Focus” clinic). These initial 14,541 pregnancies resulted in a total of 14,676 fetuses, culminating in 14,062 live births. 13,988 of these children were alive at 1 year of age. These participants were followed throughout pregnancy and they, their partners and their offspring throughout subsequent years. The collection of information continued with bolstering of the initial sample, with those who were eligible but who had not enrolled during pregnancy, taking place from the age of 7 years. The total sample size, therefore, for analyses using any data collected after age 7 is 15,454 pregnancies, resulting in 15,589 fetuses, of which 14,901 were alive at 1 year of age ^6^. Data were collected using a variety of methods including questionnaires completed by mothers, their partners and offspring; analyses of biological samples; linkage to standard data sets, and hands-on examinations including anthropometrical measures ^7, 8^.

From the age of 22, study data were collected and managed using REDCap electronic data capture tools hosted at the University of Bristol. REDCap (Research Electronic Data Capture) is a secure, web-based software platform designed to support data capture for research studies ^9^.

The study website contains details of all the data that are available through a fully searchable data dictionary and variable search tool: http://www.bristol.ac.uk/alspac/researchers/our-data/.

### Ethical approval

Ethical approval for the study was obtained from the ALSPAC Ethics and Law Committee (ALEC; IRB00003312) and the Local Research Ethics Committees. Detailed information on the ways in which confidentiality of the cohort is maintained may be found in the book by Birmingham ^10^ and on the study website: http://www.bristol.ac.uk/alspac/researchers/research-ethics/

All methods were performed in accordance with the relevant guidelines and regulations. Informed consent for the use of data collected via questionnaires and clinics was obtained from participants following the recommendations of the ALSPAC Ethics and Law Committee at the relevant time.

### Nomenclature used

**Figure 1.**
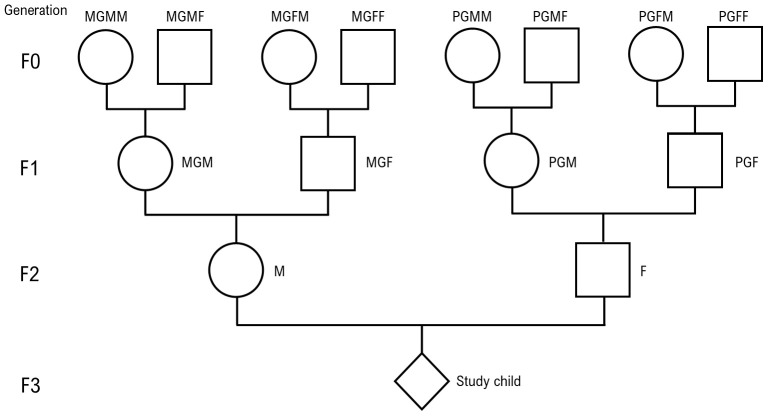
Family structure with nomenclature used (see text in Methods section). This figure has been reproduced with permission from [Golding *et al.* 2022] ^5^ ( Creative Commons Attribution 4.0 International license (CC-BY 4.0)).

The ways in which we refer to the ancestors are shown in Figure 1. The four ancestors on the maternal side of generation F0 are referred to as MGMM (maternal grandmother’s mother), MGMF (maternal grandmother’s father), MGFM (maternal grandfather’s mother) and MGFF (maternal grandfather’s father). The paternal side of generation F0 are labelled PGMM, PGMF, PGFM and PGFF, where P = paternal. For the F1 generation, the labels are MGM and MGF on the maternal side and PGM and PGF on the paternal side. F2 is represented by M (mother) and F (father). F3 is the proband who is referred to as the great-grandchild, grandchild, or child depending on which generation is under consideration.

### The Exposures

 Questionnaires administered to the study mother and her partner (usually the father of the study child) elicited details of their childhood and adolescence, including the age at which they had commenced smoking regularly, together with other information on their smoking habits, and those of their parents (i.e. the study child’s grandparents (F1s). Unfortunately, smoking habits of the F1s did not include the ages at which they had started smoking. Consequently, more recently a new questionnaire was administered to those biological parents (F2) with whom the study was still in contact, to obtain further information on their parents (F1s) and grandparents (F0s), including whether they had started smoking regularly during childhood and at what age (defined as < 17 years). Questionnaires were administered online or a paper version posted for those who preferred it. Full details of the methodology and the questions asked can be found elsewhere ^11^. In brief, for each ancestor the question asked was: ‘During his/her childhood, up to age 16, did he/she start smoking regularly?’ If yes, the age at which the smoking was started (in years) was requested, with the option ‘yes but don’t know what age’. For the analyses presented here, we have included all who started smoking prior to age 17 and who were therefore smoking in adolescence.

### The Outcomes

 Total fat mass was estimated with the use of a Lunar Prodigy DXA scanner (GE Medical Systems Lunar, Madison, WI). In this analysis we have concentrated on the measurements of fat mass at ages 17 (approximating to the end of puberty) and 24 years (early adulthood). Measurements of lean mass were measured at the same times using the same equipment as a control. Both were measured on a continuous scale.

### Confounders considered

For each ancestor studied, the following were considered as potential confounders: (i) their year of birth; (ii) ethnic group (white/non-white for F1s); (iii) social class based on occupation (manual/non-manual); (iv) no. of older siblings (0/1/2+); (v) no. of younger siblings; (vi) age of ancestor at the birth of the next generation; (vii) level of education (for F1s, not F0s and coded as equivalent to O-level+/ <O-level (examinations taken at the age of 16)); (viii) whether born in England (yes/no for F0s); (ix) trend in gross domestic product (GDP) of year of birth (F1s only); (x) business cycle of year of birth (F1s only).

### Statistical analyses

The analyses were structured to take account of the different numbers of ancestors available for study. As shown in Table 1a, the numbers available for analysis ranged from 276 (for the PGFFs) to 2462 (for the MGMs). In general, the proportion of ancestors who had smoked regularly during adolescence varied from 14–17% for grandmothers and great-grandmothers, from 43–45% for grandfathers, and from 59–63% for great-grandfathers. There were fewer ancestors on the paternal side than the maternal side for whom information was available.

**Table 1a.** Numbers of F3 ancestors who were reported to have been smoking in adolescence (SIA) and whose grandchild/great-grandchild was given a DXA scan at ages 17 and/or 24.

Ancestor concerned	DXA scan at 17: no. ≤16 at smoking onset			DXA scan at 24: no. ≤16 at smoking onset			Total N at 17	Total N at 24
	All	Male	Female	All	Male	Female		
*Maternal great-grandparents*								
MGMM	211	96	115	178	63	115	1287	1104
MGMF	542	252	290	471	194	277	877	768
MGFM	152	66	86	117	48	69	913	765
MGFF	415	179	236	338	135	203	639	539
*Paternal great-grandparents*								
PGMM	81	35	46	77	30	47	520	472
PGMF	242	95	147	225	82	143	401	379
PGFM	61	25	36	49	16	33	405	357
PGFF	183	79	104	166	68	98	297	276
*Maternal grandparents*								
MGM	415	174	241	351	123	228	2462	2089
MGF	975	415	560	820	304	516	2148	1819
*Paternal grandparents*								
PGM	206	86	120	181	70	111	1223	1065
PGF	437	177	260	437	177	260	1048	924

MGM = maternal grandmother; MGF = maternal grandfather; MGMM = maternal grandmother’s mother; MGMF = maternal grandmother’s father. MGFM = maternal grandfather’s mother; MGFF = maternal grandfather’s father. PGM = paternal grandmother; PGF = paternal grandfather; PGMM = paternal grandmother’s mother; PGMF = paternal grandmother’s father. PGFM = paternal grandfather’s mother; PGFF = paternal grandfather’s father.

Power calculations were undertaken to determine the effect sizes that a particular P-value cut-point would have 80% power of showing as significant. The results are shown for age 17 in Table 1b. These demonstrate that in general one would be 80% certain of demonstrating an approximate effect size of 1.5kg as significant with a P-value cut-point of 0.05 for the maternal grandparents, but 0.20 for paternal grandparents. For maternal great-grandparents a P-value of 0.10 would identify excess fat mass of 1.8–2.3kg, whereas for paternal great-grandparents a P-value of 0.20 would only identify excess fat masses of 2.7–3.6kg. On the basis of these discrepancies, we decided to use P<0.10 and P<0.20 as significant for associations down the maternal and paternal lines respectively in order not to lose important associations.

**Table 1b.** The minimum Kg effect sizes (mean differences) that one could be 80% sure of showing as ‘significant’ according to differing P values calculated using the 'pwr.t2n.test' in the R package 'pwr'.

Ancestor concerned	P<0.01	P<0.05	P<0.10	P<0.20
*Maternal ancestors*				
MGMM	2.76	2.26	2.01	1.71
MGMF	2.50	2.05	1.82	1.55
MGFM	3.19	2.62	2.32	1.98
MGFF	2.98	2.44	2.17	1.85
*Paternal ancestors*				
PGMM	4.35	3.56	3.16	2.70
PGMF	3.68	3.01	2.67	2.28
PGFM	5.01	4.10	3.63	3.10
PGFF	4.31	3.52	3.12	2.66
*Maternal grandparents*				
MGM	1.93	1.58	1.41	1.20
MGF	1.56	1.28	1.13	0.97
*Paternal grandparents*				
PGM	2.80	2.30	2.04	1.74
PGF	2.25	1.84	1.64	1.40

MGM = maternal grandmother; MGF = maternal grandfather; MGMM = maternal grandmother’s mother; MGMF = maternal grandmother’s father. MGFM = maternal grandfather’s mother; MGFF = maternal grandfather’s father. PGM = paternal grandmother; PGF = paternal grandfather; PGMM = paternal grandmother’s mother; PGMF = paternal grandmother’s father. PGFM = paternal grandfather’s mother; PGFF = paternal grandfather’s father.

Initial analyses determined the unadjusted associations between each of the four grandparents (F1s) and the eight great-grandparents (F0s) in regard to the grandchild’s (F3) outcomes separately for (i) all grandchildren, (ii) grandsons only and (iii) granddaughters only.

For all outcomes, adjustments were made for potential confounders that contributed 0.1% or more to R ^2^ for the relevant outcome using multiple regression. The analyses were run for all grandchildren and great-grandchildren as appropriate. The analyses were then repeated with a term for the interaction between the sex of the F3 individual and whether or not the relevant ancestor had commenced smoking prior to 17 years of age.

## Results

### Grandparents’ smoking in adolescence

***Fat mass.*** The unadjusted associations between each of the grandparents who smoked regularly in adolescence and the fat mass of their grandchildren are shown in Table 2a. There were marked associations for increased fat mass for the grandchildren if a maternal or paternal grandparent had smoked regularly in adolescence; the associations at age 17 tended to be more likely to be significant at the P values we have used than those at age 24. There were no significant differences between the sexes.

**Table 2a.** Unadjusted associations between regular smoking during the adolescence of the grandparents and fat mass in their grandchildren at ages 17 and 24. (In bold are results where the P value was <0.10 for maternal ancestors and <0.20 for paternal ancestors). The numbers involved in each analysis are shown in Table 2b.

Individual	Age	All grandchildren		Grandsons		Granddaughters	
ancestor		MD [95%CI]Kg	P	MD [95%CI]Kg	P	MD [95%CI]Kg	P
*Maternal grandparents*							
MGM	17	-0.01 [-1.05, 1.04]	**0.003**	**1.94 [0.38, 3.51]**	**.015**	1.01[-0.24, 2.27]	.114
	24	-0.83 [-1.95, 0.29]	**.046**	1.16 [-0.65, 2.97]	.209	0.88 [-0.64, 2.40]	.257
MGF	17	-0.47 [-1.31, 0.37]	**<.001**	**1.34 [0.13, 2.55]**	**.030**	**1.50 [0.46, 2.54]**	**.005**
	24	-0.40 [-1.30, 0.49]	**.001**	0.77 [-0.59, 2.13]	.267	**1.77[0.52, 3.02]**	**.005**
*Paternal grandparents*							
PGM	17	**1.38[-0.01, 2.76]**	**.052**	**1.30 [-0.67, 3.27]**	**.196**	**1.19 [-0.48, 2.87]**	**.163**
	24	0.23[-1.37,1.82]	.781	1.49 [-0.90, 3.87]	.222	-0.63 [-2.70, 1.45]	.552
PGF	17	**1.69 [0.51, 2.87]**	**.005**	**1.30 [-0.67, 3.27]**	**.031**	**1.21[-0.24, 2.67]**	**.102**
	24	**0.95 [-0.37, 2.28]**	**.158**	0.45 [-1.42, 2.32]	.636	1.16 [-0.62, 2.93]	.203

MGM = maternal grandmother; MGF = maternal grandfather; PGM = paternal grandmother; PGF = paternal grandfather

 The demographic variables associated with grandchild’s fat mass are depicted for each grandparent in Table 2b. Those with R ^2^ >0.1% were included as covariates. The consequent adjusted associations are shown in Table 2c. The numbers involved in the adjusted analyses were only approximately half of the numbers in the unadjusted analyses due to missing data in the confounders. There were no adjusted associations with either of the grandmothers smoking in adolescence, but associations with the grandfather smoking in adolescence remained, especially for the paternal grandfather. There were no indications of differences in effect sizes between the sexes of the grandchildren ( Table 2d). 

**Table 2b.** The R ^2 ^% values for the potential confounders with smoking <17. Those with R ^2^ > 0.10 were taken into account in all relevant analyses concerning the grandparents. In bold are the values of the variables included in the relevant multiple regression analyses.

Grand parent	Age tested	YoB ^a^	GDP ^b^	Bus Cyc ^c^	Ethnic group	Social Class	Older siblings	Younger siblings	Education level	Age ^d^
*Fat mass*										
MGM	17	**.50**	.44	.00	.05	**.64**	**.11**	**.23**	**.48**	.09
	24	**.98**	1.00	.01	.00	**.35**	.06	**.11**	**.73**	**.37**
MGF	17	.49	**.58**	.01	.07	**.55**	.07	**.11**	**.41**	**.11**
	24	.99	**1.09**	.02	.01	**1.35**	**.26**	.08	**.63**	**.51**
PGM	17	.07	**.14**	.04	.01	**.92**	**.39**	.09	**.38**	.00
	24	.40	**.54**	.05	.06	**.23**	**.14**	.05	**.41**	**.18**
PGF	17	.13	**.19**	**.28**	.00	**.30**	.09	.01	**.28**	.00
	24	.26	**.38**	.02	.00	**.13**	.05	**.22**	**.20**	.09
*Lean mass*										
MGM	17	.21	**.21**	.02	.02	.03	**.17**	.05	**.11**	**.11**
	24	.01	.03	.02	.02	.01	**.12**	.00	.02	.00
MGF	17	.09	**.13**	**.22**	.07	.06	.01	.05	**.11**	.03
	24	.00	.01	.07	**.10**	.06	.06	.04	.00	.03
PGM	17	**.12**	.08	.02	.01	**.19**	.01	.00	.02	.03
	24	.01	.00	.00	.06	.00	**.11**	.00	.04	.00
PGF	17	**.16**	.08	.00	.04	.05	.02	**.26**	.00	.09
	24	,07	.02	.00	.04	.02	.00	.02	.03	.05

^a^Year of birth of grandparent; ^b^Gross Domestic Product of year of birth; ^c^Business cycle; ^d^Age of grandparent when parent was born. MGM = maternal grandmother; MGF = maternal grandfather; PGM = paternal grandmother; PGF = paternal grandfather

**Table 2c.** Adjusted associations between regular smoking during adolescence (<17) of grandparents and fat mass in their grandchildren (F3) at ages 17 and 24.

Ancestor F1	Age of F3	N	MD [95%CI] Kg	P	R ^2^	P _int_
*Maternal grandparents*						
MGM	17	1340	+0.88 [-0.63, 2.38]	0.254	2.11	0.660
	24	1184	+1.03 [-0.57, 2.64]	0.208	2.94	0.984
MGF	17	1080	**+1.02 [-0.20, 2.25]**	**0.100**	2.31	0.814
	24	905	+ **1.28 [-0.11, 2.66]**	**0.071**	3.01	0.718
*Paternal grandparents*						
PGM	17	509	-0.18 [-2.27, 1.90]	0.863	1.03	0.233
	24	449	-0.73 [-3.27, 1.82]	0.575	1.16	0.591
PGF	17	563	+ **1.65 [**+ **0.04, 3.26]**	**0.045**	1.93	0.793
	24	423	+ **1.55 [-0.27, 3.38]**	**0.095**	1.85	0.483

CI = confidence interval; MD = mean difference in Kg fat mass; MGM = maternal grandmother; MGF = maternal grandfather; PGM = paternal grandmother; PGF = paternal grandfather P _int_ = P value for interaction between the sexes

**Table 2d.** Adjusted associations between regular smoking during the adolescence of the grandparents and fat mass in their grandchildren at ages 17 and 24. (In bold are results where the P value was <0.10 for maternal ancestors and <0.20 for paternal ancestors).

Grandparent	MALE F3s			FEMALE F3s		
	n	MD [95%CI]	P	n	MD [95%CI]	P
*Fat mass at 17*						
MGM	590	0.94 [-1.29, 3.16]	0.408	750	0.45 [-1.33, 2.24]	0.620
MGF	468	0.48 [-1.32, 2.28]	0.601	612	0.85 [-0.60, 2.31]	0.249
PGM	222	1.12 [-1.58, 3.82]	0.414	287	-1.37 [-3.95, 1.20]	0.295
PGF	250	**1.44 [-0.55, 3.43]**	**0.155**	313	1.23 [-0.87, 3.34]	0.251
*Fat mass at 24*						
MGM	474	1.18 [-1.32, 3.68]	0.355	710	0.59 [-1.45, 2.64]	0.570
MGF	353	0.64 [-1.38, 2.66]	0.532	552	1.43 [-0.40, 3.27]	0.125
PGM	182	0.41 [-3.34, 4.16]	0.831	267	1.89 [-5.32, 1.55]	0.280
PGF	172	**2.07 [-0.55, 4.69]**	**0.120**	251	1.04 [-1.49, 3.57]	0.418

CI = confidence interval; MD = mean difference in Kg fat mass; MGM = maternal grandmother; MGF = maternal grandfather; PGM = paternal grandmother; PGF = paternal grandfather

***Lean mass.*** In complete contrast with Table 2a: (i) whereas 23 of 24 associations were positive (i.e. greater fat mass if the grandparent had started smoking by 16 years of age), only 16 of the 24 associations with lean mass were positive; (ii) whereas 15 out of 24 unadjusted mean differences in fat mass were highlighted as reaching our defined P value cut-points, only 3 of the 24 unadjusted statistics for lean mass did so ( Table 3a). 

**Table 3a.** Unadjusted associations between regular smoking in adolescence of grandparents and **lean** mass in their grandchildren at ages 17 and 24.

Individual	Age	All grandchildren		Grandsons		Granddaughters	
ancestor		MD [95%CI]Kg	P	MD [95%CI]Kg	P	MD [95%CI]Kg	P
*Maternal grandparents*							
MGM	17	-0.01 [-1.05, 1.04]	.991	0.68 [-0.32, 1.69]	.182	0.32 [-0.26, 0.90]	.274
	24	-0.83 [-1.95, 0.29]	.147	0.13 [-1.26, 1.51]	.856	-0.08[-0.85, 0.69]	.835
MGF	17	-0.47 [-1.31, 0.37]	.274	0.40 [-0.38, 1.17]	.313	-0.17 [-0.65, 0.31]	.483
	24	-0.40 [-1.30, 0.49]	.380	0.59 [-0.45, 1.63]	.264	-0.13[-0.76, 0.50]	.677
*Paternal grandparents*							
PGM	17	0.43 [-1.04, 1.90]	.569	**1.13 [-0.31, 2.57]**	**.125**	0.47 [-0.33, 1.28]	.250
	24	0.49 [-1.06, 2.94]	.536	**1.55 [-0.31, 3.41]**	**.101**	0.05 [-1.01, 1.10]	.931
PGF	17	-0.23 [-1.43, 0.97]	.707	0.04 [-1.16, 1.23]	.953	**0.45 [-.21, 1.12]**	**.179**
	24	0.24 [-1.02, 1.49]	.712	0.79 [-0.71, 2.30]	.301	0.40 [-.47, 1.27]	.365

CI = confidence interval; MD = mean difference in Kg fat mass; MGM = maternal grandmother; MGF = maternal grandfather; PGM = paternal grandmother; PGF = paternal grandfather

Interestingly, very few of the socioeconomic and demographic variables were associated with lean mass, compared with fat mass ( Table 2b). For example, the social class and education levels of each of the grandparents contributed to the grandchild’s fat mass, whereas this only occurred rarely for lean mass. Adjustment for these potential confounders showed little of interest ( Table 3b) apart from an interaction with the sex of the grandchild if the PGM had smoked in adolescence (with increased effect size among 24-year-old grandsons compared to granddaughters) ( Table 3c).

**Table 3b.** **Adjusted** associations between regular smoking during adolescence (<17) of grandparents and **lean** mass in their **grandchildren** at ages 17 and 24.

Ancestor F1	Age of F3	N	MD [95%CI] Kg	P	R ^2^	P _int_
*Maternal grandparents*						
MGM	17	1502	0.48 [-0.93, 1.88]	0.506	0.71	0.623
	24	1735	-0.60 [-1.84, 0.65]	0.346	0.11	0.476
MGF	17	1472	0.27 [-0.80, 1.34]	0.621	0.86	0.147
	24	1711	-0.26 [-1.19, 0.66]	0.574	0.06	0.142
*Paternal grandparents*						
PGM	17	502	0.79 [-1.60, 3.17]	0.518	1.64	0.384
	24	856	0.82 [-0.87, 2.50]	0.342	0.24	**0.090**
PGF	17	607	-0.20 [-2.04, 1.30]	0.662	0.31	0.223
	24	924	0.24 [-1.02, 1.49]	0.712	0.01	0.636

CI = confidence interval; MD = mean difference in Kg fat mass; MGM = maternal grandmother; MGF = maternal grandfather; PGM = paternal grandmother; PGF = paternal grandfather P _int _= P value for interaction between the sexes

**Table 3c.** **Adjusted** associations between regular smoking during adolescence of the grandparents (F1) and **lean** mass in their grandchildren at ages 17 and 24. (In bold are results where the P value was <0.10 for maternal ancestors and <0.20 for paternal ancestors).

Grandparent	GRANDSONS			GRANDDAUGHTERS		
	n	MD [95%CI]	P	N	MD [95%CI]	P
*Lean mass at 17*						
MGM	652	0.94 [-0.45, 2.33]	0.186	850	0.51 [-0.25, 1.27]	0.187
MGF	654	**0.96 [-0.05, 1.96]**	**0.063**	818	0.22 [-0.36, 0.79]	0.461
PGM	219	**1.79 [-0.54, 4.12]**	**0.132**	283	0.36 [-1.04, 1.77]	0.611
PGF	272	-0.38 [-1.88, 1.12]	0.619	335	0.51 [-0.45, 1.47]	0.298
*Lean mass at 24*						
MGM	695	0.48 [-1.03, 1.98]	0.533	1040	-0.24 [-1.10, 0.62]	0.584
MGF	661	0.69 [-0.39, 1.77]	0.210	1050	-0.19 [-0.83, 0.45]	0.561
PGM	331	**2.10 [0.07, 4.14]**	**0.043**	523	0.08 [-1.08, 1.25]	0.889
PGF	358	0.79 [-0.71, 2.30]	0.301	566	0.40 [-0.47, 1.27]	0.365

CI = confidence interval; MD = mean difference in Kg fat mass; MGM = maternal grandmother; MGF = maternal grandfather; PGM = paternal grandmother; PGF = paternal grandfather

### Great-grandparents’ smoking in adolescence

***Fat mass.*** The unadjusted associations between the great-grandparents’ age <17 at smoking regularly and fat mass of the great-grandchildren is shown in Table 4a. When the maternal great-grandparents had smoked in adolescence, their great-grandchildren tended to have more fat mass on average, with the exception of the great-grandchildren of the MGFF’s, where the associations were negative. The only associations at P<0.10 concerned an excess of fat mass at age 17 if the MGMM had smoked regularly in adolescence; there was no such association at age 24. For paternal grandparents, there were four associations at P<0.20, each involving the 24-year-olds.

**Table 4a.** Unadjusted associations between regular smoking in adolescence (<17) of great-grandparents and fat mass in their great-grandchildren at ages 17 and 24. Data shown comprise the mean differences (MD) between the fat mass of the great-grandchildren of those great-grandparents who smoked <17 compared with the rest of the population.

Individual	age	All		Males		Females	
		MD [95%CI]Kg	P	MD [95%CI]Kg	P	MD [95%CI]Kg	P
*Maternal great-grandparents*							
MGMM	17	**2.06 [0.60, 3.52]**	**.006**	**2.72 [0.70, 4.73]**	**.008**	**1.62 [-0.15, 3.39]**	**.073**
	24	1.16 [-0.48, 2.80]	.164	1.42 [-1.17, 4.00]	.281	0.63 [-1.44, 2.69]	.550
MGMF	17	0.57 [-0.72, 1.86]	.386	0.71 [-1.03, 2.46]	.423	0.53 [-1.05, 2.10]	.512
	24	1.08 [-0.32, 2.48]	.130	1.15 [-0.92, 3.21]	.276	1.04 [-0.80, 2.88]	.267
MGFM	17	0.57 [-1.13, 2.28]	.509	1.68 [-0.62, 3.98]	.151	-0.46 [-2.58, 1.65]	.667
	24	0.39 [-1.62, 2.40]	.700	0.12 [-2.65, 2.89]	.934	0.67 [-0.21, 3.38]	.630
MGFF	17	-0.17 [-1.69, 1.35]	.831	-0.16 [-2.38, 2.07]	.889	-0.30 [-2.15, 1.56]	.754
	24	-1.15 [-2.88, 0.58]	.192	-0.26 [-2.89, 2.37]	.845	-1.50 [-3.74, 0.73]	.186
*Paternal great-grandparents*							
PGMM	17	0.36 [-1.89, 2.60]	.754	1.33 [-1.94, 4.60]	.423	-0.33 [-3.00, 2.34]	.808
	24	-0.96 [-3.36, 1.44]	.432	0.81 [-3.07, 4.68]	.682	**-2.01 [-5.03, 1.02]**	**.192**
PGMF	17	1.86 [-0.96, 4.68]	.209	-0.01[-2.84, 2.82]	.994	1.21 [-0.87, 3.29]	.254
	24	0.33 [-1.60, 2.26]	.734	**-2.23 [-5.29, 0.84]**	**.153**	**1.74 [-0.71, 4.19]**	**.162**
PGFM	17	0.55 [-1.95, 3.04]	.608	2.18 [-1.49,5.84]	.243	-0.96 [-3.95, 2.03]	.529
	24	0.31 [-2.53, 3.16]	.828	2.42 [-2.83,7.66]	.363	-0.94 [-4.27, 2.39]	.578
PGFF	17	1.60 [-1.68,4.88]	.337	.85 [-3.76, 5.45]	.717	1.39 [-2.59, 5.38]	.491
	24	1.94 [-1.65,5.54]	.289	**5.47 [-.52, 1.15]**	**.073**	0.10 [-4.57, 4.37]	.966

CI = confidence interval; MD = mean difference in Kg fat mass MGMM = maternal grandmother’s mother; MGMF = maternal grandmother’s father. MGFM = maternal grandfather’s mother; MGFF = maternal grandfather’s father. PGMM = paternal grandmother’s mother; PGMF = paternal grandmother’s father. PGFM = paternal grandfather’s mother; PGFF = paternal grandfather’s father.

On adjustment for the demographic variables ( Table 4b), two of the 16 associations reached the P value stipulated in advance (P<0.10); both associations were negative and were related to the 24-year-olds (involving the MGFM and MGFF). This number of significant adjusted associations was no greater than would have been expected by chance. Similarly, examination of the 32 associations considering the sexes separately, revealed only five below the P value cut-off, and none exhibited consistency between the two age groups ( Table 4c and Table 4d). 

**Table 4b.** The R ^2 ^% values for the potential confounders in regard to the great-grandparents. Those with R ^2^ > 0.10 were taken into account in all relevant analyses concerning the great-grandparents. In bold are the values of the variables included in the relevant multiple regression analyses.

Great- grandparent	Age at Measure	Resident In England ^a^	Year of birth	Older siblings	Younger siblings	Social class	Age at Delivery ^b^
*Fat mass*							
MGMM	17	.08	**.46**	.03	**.21**	.03	**.21**
	24	.02	**1.77**	.09	**.55**	**.23**	**.36**
MGMF	17	.02	**.42**	.05	**.25**	.02	.00
	24	.03	**.83**	**1.47**	.07	**.37**	.03
MGFM	17	**.23**	**.40**	**.69**	. **45**	**.13**	.01
	24	**.17**	**1.00**	.02	.00	.00	.00
MGFF	17	**.13**	**.17**	.00	.05	.00	.06
	24	**.51**	**.57**	.02	.00	**.60**	.00
PGMM	17	.00	**.88**	.00	.06	**.93**	**.72**
	24	**.65**	**.81**	**.12**	.09	**.33**	**.62**
PGMF	17	**.13**	**.25**	**.77**	.63	**.15**	**.16**
	24	**.75**	**.99**	.08	**.40**	**.69**	**.30**
PGFM	17	.03	**.22**	.02	**.25**	**.10**	.00
	24	.02	**1.89**	**.20**	**.28**	**.33**	**.78**
PGFF	17	**.10**	**.27**	**1.21**	.06	**.16**	.00
	24	.06	**2.22**	**.30**	.00	**1.02**	**2.06**
*Lean mass*							
MGMM	17	.08	**.46**	.03	**.21**	.03	**.21**
	24	.07	**.13**	.03	.01	**.22**	.02
MGMF	17	.02	**.42**	.05	**.25**	.02	.00
	24	.00	**.21**	**.30**	.01	.00	.01
MGFM	17	**.23**	**.40**	**.69**	**.45**	**.13**	.00
	24	**.34**	**.11**	**.33**	**.34**	.01	.01
MGFF	17	**.13**	**.17**	.00	.05	.00	.06
	24	**.15**	.02	**.30**	.00	.02	.00
PGMM	17	.00	**.88**	.00	.06	**.93**	**.72**
	24	**.46**	**.36**	.06	.03	**.25**	.00
PGMF	17	**.13**	**.25**	**.77**	**.63**	**.15**	**.16**
	24	.02	.00	**.95**	.03	.02	.00
PGFM	17	.03	**.22**	.02	**.25**	**.10**	.01
	24	.05	**.33**	.04	**.18**	**.83**	.08
PGFF	17	**.10**	**.27**	**1.21**	.06	**.16**	.00
	24	**.51**	.02	**.24**	.00	.00	**.11**

^a^Great-grandparent was in England when born; ^b^Age when relevant grandparent was born. MGMM = maternal grandmother’s mother; MGMF = maternal grandmother’s father. MGFM = maternal grandfather’s mother; MGFF = maternal grandfather’s father. PGMM = paternal grandmother’s mother; PGMF = paternal grandmother’s father. PGFM = paternal grandfather’s mother; PGFF = paternal grandfather’s father.

**Table 4c.** Adjusted associations between regular smoking in adolescence (<17) of great-grandparents (F0) and fat mass in their great-grandchildren (F3) at ages 17 and 24. Data shown comprise the mean differences (MD) between the **fat** mass of the great-grandchildren of those great-grandparents who smoked <17 compared with the rest of the population.

Ancestor F0	Age of F3	N	MD [95%CI]Kg	P	R ^2^	P _int_
*Maternal great-grandparents*						
MGMM	17	634	1.81 [-0.42, 4.04]	0.111	3.12	0.558
	24	563	0.66 [-1.88, 3.19]	0.611	3.41	0.927
MGMF	17	386	-0.41 [-2.36, 1.54]	0.679	3.71	0.763
	24	317	-0.88 [-3.05, 1.29]	0.425	4.89	0.439
MGFM	17	229	-0.54 [-3.86, 2.77]	0.748	6.56	0.509
	24	467	**-3.48 [-6.21, -0.74]**	**0.013**	2.49	0.547
MGFF	17	372	0.43 [-1.54, 2.40]	0.667	1.04	0.763
	24	282	**-2.00 [-4.26, 0.26]**	**0.082**	2.30	0.934
*Paternal great-grandparents*						
PGMM	17	186	-2.74 [-0.63,2.38]	0.254	2.11	0.660
	24	123	-2.45 [-8.96, 4.05]	0.457	3.45	0.853
PGMF	17	232	1.35 [-1.13, 3.84]	0.285	3.02	0.480
	24	139	0.17 [-3.30, 3.64]	0.924	4.91	**0.140**
PGFM	17	86	-1.23 [-6.60, 4.13]	0.649	1.93	0.823
	24	82	-2.38 [-8.83, 4.08]	0.466	2.90	0.596
PGFF	17	102	1.75 [-1.82, 5.32]	0.333	17.1	0.948
	24	102	0.05 [-3.60, 3.70]	0.980	13.4	0.886

MD = mean difference in Kg fat mass; MGMM = maternal grandmother’s mother; MGMF = maternal grandmother’s father. MGFM = maternal grandfather’s mother; MGFF = maternal grandfather’s father. PGMM = paternal grandmother’s mother; PGMF = paternal grandmother’s father. PGFM = paternal grandfather’s mother; PGFF = paternal grandfather’s father. P _int_ = P value for interaction between the sexes

**Table 4d.** Adjusted associations between regular smoking during adolescence of the great-grandparents (F1) and fat mass in their great-grandchildren (F3) at ages 17 and 24. (In bold are results where the P value was <0.10 for maternal ancestors and <0.20 for paternal ancestors).

Great-Grandparent	MALE F3s			FEMALE F3s		
	n	MD[95%CI]	P	n	MD[95%CI]	P
*Fat mass at 17*						
MGMM	287	**3.43 [0.59, 6.27]**	**0.018**	347	1.68 [-1.15, 4.50]	0.244
MGMF	181	0.37 [-1.91, 2.64]	0.751	205	0.94 [-1.59, 3.46]	0.466
MGFM	110	0.64 [-3.86, 5.14]	0.779	119	-1.70 [-5.60, 2.20]	0.389
MGFF	174	1.07 [-1.70, 3.84]	0.447	198	0.03 [-2.38, 2.44]	0.980
PGMM	81	-0.94 [-7.02, 5.14]	0.760	105	**-4.23 [-8.64, 0.19]**	**0.060**
PGMF	58	1.76 [-1.93, 5.45]	0.343	86	0.43 [-1.33, 2.18]	0.629
PGFM	30	-0.05 [-6.22, 6.12]	0.986	56	**2.45 [-0.35, 5.25]**	**0.086**
PGFF	39	-0.96 [-5.47, 3.55]	0.668	62	-0.05 [-1.92, 1.81]	0.953
*Fat mass at 24*						
MGMM	232	0.11 [-3.71, 3.93]	0.956	331	0.71 [-2.56, 3.97]	0.671
MGMF	141	0.49 [-3.04, 4.02]	0.784	176	-1.30 [-3.98, 1.39]	0.342
MGFM	197	**-4.44 [-8.29, -0.59]**	**0.024**	270	-2.92 [-6.65, 0.80]	0.123
MGFF	126	-1.61 [-4.70, 1.49]	0.307	156	-2.51 [-5.62, 0.61]	0.114
PGMM	48	-3.14 [-14.8, 8.51]	0.589	75	-4.78[-12.9, 3.31]	0.242
PGMF	55	-2.36 [-7.57, 2.86]	0.369	84	1.65 [-3.21, 6.51]	0.501
PGFM	27	-3.16 [-31.3, 25.0]	0.817	55	**-4.17 [-9.53, 1.19]**	**0.125**
PGFF	38	0.78 [-6.55, 8.11]	0.829	64	-0.33 [-4.71, 4.05]	0.880

CI = confidence interval; MD = mean difference in Kg fat mass MGMM = maternal grandmother’s mother; MGMF = maternal grandmother’s father. MGFM = maternal grandfather’s mother; MGFF = maternal grandfather’s father. PGMM = paternal grandmother’s mother; PGMF = paternal grandmother’s father. PGFM = paternal grandfather’s mother; PGFF = paternal grandfather’s father.

***Lean mass.*** Of the 48 unadjusted associations, only five reached an appropriate P value – i.e. no more than would be expected by chance. On adjustment, two of the 16 comparisons reached a relevant P value, again no more than expected ( Table 5a– Table 5c).

**Table 5a.** Unadjusted associations between regular smoking in adolescence of great-grandparents and **lean** mass in their **great-grandchildre** **n** at ages 17 and 24. Data shown comprise the mean differences (MD) between the lean mass of the F3s of those great-grandparents smoking <17 and the rest of the population.

Individual	Age	All		Males		Females	
		MD[95%CI]Kg	P	MD[95%CI]Kg	P	MD[95%CI]Kg	P
*Maternal great-grandparents*							
MGMM	17	0.78 [-0.66, 2.23]	.289	0.77 [-0.63, 2.16]	.280	0.58 [-0.24, 1.40]	.163
	24	-0.37 [-1.91, 1.16]	.633	0.83 [-1.11, 2.76]	.401	0.41 [-0 .62, 1.44]	.434
MGMF	17	0.51 [-0.85, 1.87]	.461	0.48 [-0.79, 1.75]	.456	0.37 [-0.34, 1.08]	.307
	24	0.50 [-0.88, 1.88]	.476	1.00 [-0.57, 2.57]	.213	0.13 [-0.80, 1.05]	.786
MGFM	17	-0.10 [-1.84, 1.63]	.905	0.87 [-0.86, 2.60]	.321	-0.49 [-1.45, 0.47]	.318
	24	0.60 [-1.27, 2.46]	.530	0.69 [-1.44, 2.82]	.522	0.26 [-1.15, 1.68]	.714
MGFF	17	-0.31 [-1.94, 1.33]	.711	0.51 [-0.99, 2.01]	.505	-0.62 [-0.15, 0.27]	.173
	24 *	-0.19 [-1.92, 1.54]	.830	0.68 [-1.34, 2.69]	.508	**-1.54 [-2.64, -0.44]**	**.006**
*Paternal great-grandparents*							
PGMM	17	0.77 [-1.59, 3.14]	.522	-0.22 [-2.42, 1.97]	.842	**1.41 [0.22, 2.59]**	**.020**
	24	0.95 [-1.44, 3.34]	.437	0.84 [-1.99, 3.66]	.560	0.63 [-0.95, 2.21]	.435
PGMF	17	-0.76 [-2.76, 1.24]	.454	0.38 [-1.53, 2.29]	.683	0.41 [-0.64, 1.46]	.440
	24	-0.31 [-2.35, 1.72]	.762	0.28 [-2.16, 2.72]	.820	0.84 [-0.46, 2.14]	.204
PGFM	17	-1.02[-3.74, 1.70]	.463	-0.20 [-2.84, 2.44]	.882	-0.74 [-2.21, 0.73]	.323
	24	-1.45[-4.34, 1.45]	.323	0.47 [-3.09, 4.04]	.794	-0.92 [-2.79, 0.95]	.332
PGFF	17 *	1.04 [-1.23, 3.31]	.367	-1.24 [-3.27, 0.79]	.228	**1.27 [0.09, 2.44]**	**.035**
	24	**1.83 [-0.46, 4.11]**	**.116**	0.26 [-2.23, 2.75]	.837	**1.88 [0.36, 3.39]**	**.015**

MD = mean difference lean mass in Kg; * interaction between sexes CI = confidence interval; MD = mean difference in Kg fat mass MGMM = maternal grandmother’s mother; MGMF = maternal grandmother’s father. MGFM = maternal grandfather’s mother; MGFF = maternal grandfather’s father. PGMM = paternal grandmother’s mother; PGMF = paternal grandmother’s father. PGFM = paternal grandfather’s mother; PGFF = paternal grandfather’s father.

**Table 5b.** Adjusted associations between regular smoking in adolescence (<17) of great-grandparents and **lean** mass in their **great-grandchildren** at ages 17 and 24. Data shown comprise the mean differences (MD) between the fat mass of the great-grandchildren of those great-grandparents who smoked regularly <17 compared with the rest of the population.

Ancestor F0	Age of great- grandchild	N	MD [95%CI]Kg	P	R ^2^	P _int_
*Maternal great-grandparents*						
MGMM	17	634	**2.27 [0.07, 4.46]**	**0.043**	1.66	0.298
	24	498	0.16 [-2.25, 2.57]	0.896	0.53	0.264
MGMF	17	386	**2.36 [0.38, 4.35]**	**0.020**	2.53	0.434
	24	351	1.43 [-0.61, 3.48]	0.169	0.90	0.671
MGFM	17	229	-1.21 [-4.99, 2.56]	0.528	2.32	0.406
	24	305	-2.18 [-5.45, 1.09]	0.191	3.19	0.642
MGFF	17	372	0.75 [-1.43, 2.93]	0.497	0.90	0.869
	24	263	1.35 [-1.14, 3.85]	0.287	0.91	**0.034**
*Paternal great-grandparents*						
PGMM	17	186	-0.70 [-5.12, 3.72]	0.754	0.85	0.936
	24	175	-0.10 [-4.44, 4.24]	0.964	1.65	0.423
PGMF	17	144	0.83 [-2.53, 4.19]	0.625	3.17	0.386
	24	182	-0.71 [-3.59, 2.17]	0.626	0.42	0.884
PGFM	17	86	2.03 [-3.60, 7.66]	0.475	3.47	0.399
	24	88	-2.62 [-8.51, 3.27]	0.379	3.53	0.967
PGFF	17	101	0.14 [-3.99, 4.27]	0.946	8.99	0.393
	24	109	0.84 [-3.13, 4.80]	0.676	1.28	0.744

MD = mean difference in Kg fat mass; MGMM = maternal grandmother’s mother; MGMF = maternal grandmother’s father. MGFM = maternal grandfather’s mother; MGFF = maternal grandfather’s father. PGMM = paternal grandmother’s mother; PGMF = paternal grandmother’s father. PGFM = paternal grandfather’s mother; PGFF = paternal grandfather’s father. P _int _= P value for interaction between the sexes

**Table 5c.** Adjusted associations between onset of regular smoking during adolescence of the great-grandparents (F1) and **lean** mass in their great-grandchildren (F3) at ages 17 and 24. (In bold are results where the P value was <0.10 for maternal ancestors and <0.20 for paternal ancestors).

Great-Grandparent	GREAT-GRANDSONS			GREAT-GRANDDAUGHTERS		
	N	MD [95%CI]	P	n	MD [95%CI]	P
*Lean mass at 17*						
MGMM	287	**1.79 [-0.28, 3.86]**	**0.090**	347	0.60 [-0.72, 1.92]	0.373
MGMF	181	0.04 [-1.81, 1.88]	0.969	205	**0.88 [-0.17, 1.93]**	**0.100**
MGFM	110	-0.10 [-3.46, 3.25]	0.951	119	-1.36 [-3.68, 0.95]	0.247
MGFF	174	0.70 [-1.21, 2.61]	0.472	198	0.33 [-0.88, 1.54]	0.593
PGMM	81	-0.94 [-7.02, 5.14]	0.760	105	**-4.23 [-8.64, 0.19]**	**0.060**
PGMF	93	-0.33 [-4.25. 3.58]	0.867	139	1.57 [-1.34, 4.47]	0.288
PGFM	30	0.32 [-9.73, 10.4]	0.948	56	-0.96 [-6.45, 4.52]	0.725
PGFF	40	1.08 [-5.04, 7.19]	0.722	62	1.03 [-3.52, 5.59]	0.651
*Lean mass at 24*						
MGMM	203	-0.35 [-3.34, 2.63]	0.815	295	1.20 [-0.42, 2.82]	0.145
MGMF	154	0.66 [-1.66, 2.98]	0.574	197	0.01 [-1.31, 1.34]	0.987
MGFM	134	-1.00 [-5.04, 3.04]	0.624	171	-1.38 [-3.64, 0.88]	0.229
MGFF	110	1.81 [-1.13, 4.74]	0.226	153	**-1.56 [-3.08, -0.05]**	**0.043**
PGMM	81	-0.98 [-5.62, 3.66]	0.674	105	-0.34 [-2.35, 1.67]	0.736
PGMF	58	1.76 [-1.93, 5.45]	0.343	86	0.43 [-1.33, 2.18]	0.629
PGFM	30	-0.05 [-6.22, 6.12]	0.986	56	**2.45 [-0.35, 5.25]**	**0.086**
PGFF	39	-0.96 [-5.47, 3.55]	0.668	62	-0.05 [-1.92, 1.81]	0.953

CI = confidence interval; MD = mean difference in Kg fat mass MGMM = maternal grandmother’s mother; MGMF = maternal grandmother’s father. MGFM = maternal grandfather’s mother; MGFF = maternal grandfather’s father. PGMM = paternal grandmother’s mother; PGMF = paternal grandmother’s father. PGFM = paternal grandfather’s mother; PGFF = paternal grandfather’s father.

## Discussion

 Our research aim has been to ascertain whether exposure to an environmental insult such as regular smoking in the adolescence of ancestors had any discernible consequences on fat mass in the grandchildren and/or great-grandchildren. We used lean mass effects as a contrast, to ensure that any effect of fat mass was not true of any other anthropometric measure. Based on both the Ӧverkalix studies ^1^, and our earlier findings of an association between pre-pubertal onset of paternal smoking and increased fat mass in sons, but not daughters ^3^, we showed in an earlier study that there were sex-specific effects on grandchildren and great-grandchildren if their ancestor had commenced regular smoking pre-puberty ^5^. Despite small numbers and wide confidence intervals, we found that there was evidence of increased fat mass in granddaughters and great-granddaughters at ages17 and 24, associated with ancestors who commenced smoking pre-puberty (<13 years) compared with those who commenced in adolescence (aged 13–16). No such associations were noted with lean mass.

In this set of analyses, we have assessed whether there were associations between the amount of fat mass in the grandchildren and great-grandchildren of men and women who had smoked regularly in adolescence compared with the rest of their peers. We have shown here associations between the grandfathers smoking in adolescence and the fat mass of their grandchildren, and that this was apparent for the grandchildren in both their late teens and early adulthood (ages 17 and 24), and for both the maternal and paternal lines, contrary to our hypothesis. There were no such associations if either of the grandmothers had smoked in adolescence. There were no convincing associations between the great-grandparents smoking in adolescence and the fat or lean mass of their great-grandchildren.

Previous analyses have stressed the importance of the timing of exposures in regard to outcomes in succeeding generations. We have shown this in regard to exposures *in utero* as well as in the pre-puberty period, with apparent effects on outcomes as diverse as autistic traits, myopia, obesity and IQ ^12^. Here we have demonstrated an association with an exposure to cigarette smoking in the adolescent period and suggest that this period of time should also be considered in further multi-generational studies. However, it should be noted that, unlike the associations with pre-pubertal smoking, there was no indication of any consistent associations in the great-grandchildren, indicating that the associations may indicate intergenerational rather than transgenerational inheritance. 

This study has a number of weaknesses: (a) the data on age at onset of regular smoking of their ancestors was obtained retrospectively from their children and grandchildren. Although there is anecdotal evidence that ancestors who started smoking pre-adolescence are prone to remember and even boast about this, it is unclear as to whether those starting smoking at later ages (i.e. age 13–16) were as likely to recall such detail. (b) There was a large amount of information missing on age at onset of smoking; we did not try to impute these data since we were unsure whether they were missing at random. Consequently, the adjusted analyses were carried out with complete data only, with obvious reduction in statistical power, particularly for the paternal line. To compensate for this, and to ensure that we did not ignore relevant associations, we considered P values <0.10 for the maternal line and <0.20 for the paternal line. (c) We were not able to replicate our findings as we are not aware of any other studies with similar relevant data.

The strengths of the study lie in: (i) its longitudinal nature; (ii) the fact that outcomes used the DXA measures of fat and lean mass, which are considerably more accurate than indicators such as BMI (body mass index) which do not distinguish between fat, lean or bone mass, and (iii) the associations we demonstrated were apparent for the two ages tested.

In conclusion, our research question concerned whether exposures to cigarette smoking in the age group 13–16 years compared with not starting smoking until age 17 or later, or not at all, was associated with outcomes in the grandchildren or great-grandchildren. We have shown here that exposures to cigarette smoking at this age by the grandfather, but not the grandmother, were associated with fat, but not lean, body mass. The fact that no such effects were found among the great-grandchildren may indicate that the associations are intergenerational rather than transgenerational. Alternatively, it may indicate weakening of effects across generations possibly obscured by a multitude of other factors. Clearly further longitudinal family studies are important in order to assess whether these results are generalisable.

## Data availability

ALSPAC data access is through a system of managed open access. The steps below highlight how to apply for access to the data included in this Data Note and all other ALSPAC data:

1. Please read the ALSPAC access policy which describes the process of accessing the data and samples in detail, and outlines the costs associated with doing so.

2. You may also find it useful to browse our fully searchable research proposal database which lists all research projects that have been approved since April 2011.

3. Please submit your research proposal for consideration by the ALSPAC Executive Committee. You will receive a response within 10 working days to advise you whether your proposal has been approved.

If you have any questions about accessing data, please email alspac-data@bristol.ac.uk.

The Study website also contains details of all the data that is available through a fully searchable data dictionary.
