# Supplementary material for: Regular smoking of male ancestors in adolescence and fat mass in young adult grandchildren and great-grandchildren
Source: Wellcome Open Res. 2023 Mar 23;7:184. Originally published 2022 Jul 8. [Version 2] doi: 10.12688/wellcomeopenres.17950.2 (PMC9597126; doi:10.12688/wellcomeopenres.17950.2)
Supplement: Peer review history

# Peer review: Reviewer response for version 2

**DOI:** 10.21956/wellcomeopenres.21081.r55711

**Published:** 2023-03-27

**Authors:** Anthony P. Monaco

**Affiliations:** 1 Tufts University, Medford, MA, USA

The authors have revised the manuscript and addressed the reviewers' concerns.

Is the work clearly and accurately presented and does it cite the current literature?

Partly

If applicable, is the statistical analysis and its interpretation appropriate?

I cannot comment. A qualified statistician is required.

Are all the source data underlying the results available to ensure full reproducibility?

Yes

Is the study design appropriate and is the work technically sound?

Yes

Are the conclusions drawn adequately supported by the results?

Yes

Are sufficient details of methods and analysis provided to allow replication by others?

Yes

Reviewer Expertise:

Human genetics, epigenetics

I confirm that I have read this submission and believe that I have an appropriate level of expertise to confirm that it is of an acceptable scientific standard.

# Peer review: Reviewer response for version 1

**DOI:** 10.21956/wellcomeopenres.19893.r54462

**Published:** 2023-02-09

**Authors:** Gemma Lewis

**Affiliations:** 1 Division of Psychiatry, Faculty of Brain Sciences, University College London, London, UK

This study uses the large ALSPAC cohort to investigate associations between smoking among ancestors and ‘fat mass’ in their grandchildren. The main finding was a positive association between adolescent smoking by grandfathers and increased ‘fat mass’ among their young adult grandchildren. The authors explained the complex design clearly and the ALSPAC cohort is large, with a rich amount of novel data. The long follow-up into young adulthood is also a strength. I have several suggestions to improve the manuscript:

 The hypotheses were clearly stated. However, the introduction would benefit from a summary of the theoretical (not just empirical) justification of the hypotheses proposed: what is the theory underlying/leading to these hypotheses, and why are they plausible in terms of potential mechanisms/explanations?

 Throughout the manuscript, the authors could tone down strongly causal language which seems inconsistent with the observational design (e.g. effects, determined, consequences, ascertain etc).

 In the introduction, the authors could consider replacing the term ‘fatter’ with a more medical objective term.

 In the introduction there was a good critical appraisal of existing evidence but I didn’t get a strong sense of the broader context/bigger picture: why is this research question important; where would it lead in terms of advancements / implications?

 The discussion would benefit from a section on potential mechanisms that underlie these associations. Why do the authors think that this patter of associations was observed in terms of underlying mechanisms?

 The discussion would also benefit from consideration of the potential practical implications of these findings.

 The limitations section could be expanded upon. I wasn’t convinced by the anecdotal evidence that people boast about smoking pre-puberty and thought that could be removed. The authors could justify their assumption/uncertainty about whether data were MAR. The large amount of systematic attrition across follow-up is a limitation of the ALSPAC cohort, and strategies to replace missing data (such as MI and FIML) are commonly used and recommended. I think that lack of a strategy to address missing data (particularly in the outcomes due to attrition to adulthood) is one of the main limitations of the study. If not addressed analytically, this should be discussed in more detail. It isn’t just the reduction in power that is a potential problem – the risk of bias due to missing data should also be addressed/discussed. The limitations section should also cover the possibility of residual confounding, which can never be ruled out in observational studies. Some of the confounders – mainly SES – were measured quite crudely (manual, non-manual) and this could be discussed. Genetic confounding of these associations also seems possible.

 There are a lot of Tables and it would the manuscript easier to digest if some of these were moved to the supplement (perhaps the ones not containing main findings).

 The paper takes a binary approach to interpreting p values, which are instead a continuous construct. Could the authors reconsider this? I thought ALSPAC guidance was to avoid the binary terms ‘significant’ and ‘non-significant’ which encourage over reliance on an arbitrary binary threshold. I would reconsider the related strategy of emboldening ‘significant’ findings.

 The authors select confounders based on the R squared value of the confounder. This is an unusual approach in epidemiology. I think the authors should either reconsider this strategy or justify their approach in more detail, with supporting evidence/references etc. Usually, a confounder would be included if it altered the association between exposure and outcome. Reliance upon R squared is a strategy more suitable for predictive modelling. It also does not explore whether the confounder is associated with the exposure (as well as the outcome).

 The regression modelling could be explained more clearly

 In the section on data availability, I think the authors should mention the financial costs required to access ALSPAC. Definitions of open access vary but one is: Open access is a set of principles and a range of practices through which research outputs are distributed online, free of access charges or other barriers. Could the authors consider whether the ALSPAC data are in fact open access to researchers outside of Bristol University (because of the financial costs)?

Is the work clearly and accurately presented and does it cite the current literature?

Partly

If applicable, is the statistical analysis and its interpretation appropriate?

Partly

Are all the source data underlying the results available to ensure full reproducibility?

Partly

Is the study design appropriate and is the work technically sound?

Yes

Are the conclusions drawn adequately supported by the results?

Partly

Are sufficient details of methods and analysis provided to allow replication by others?

Partly

Reviewer Expertise:

Psychiatric epidemiology

I confirm that I have read this submission and believe that I have an appropriate level of expertise to confirm that it is of an acceptable scientific standard, however I have significant reservations, as outlined above.

# Peer review: Reviewer response for version 1

**DOI:** 10.21956/wellcomeopenres.19893.r52588

**Published:** 2022-10-25

**Authors:** Alison Holloway

**Affiliations:** 1 Department of Obstetrics and Gynecology, McMaster University, Hamilton, ON, Canada

The strength of this work is that it is based on the ALSPAC longitudinal study which has collected information regarding smoking across multiple generations. The authors have looked at the timing of smoking initiation in great-grandparents and grandparents and the association with fat mass in the study child (F3). They have attempted to control for a number of cofounding factors; whether or not there are more than is justified by the sample size, is a question for a statistical expert.

 While the authors make conclusions about the impact of ancestral smoking it is somewhat unclear how they have dealt with the fact that there seems to be more than one observation per F0 ancestor. For example, according to table 1 there are a total of 389 MGMM subjects but 1289 grandchildren/great grandchildren. How did the authors deal with multiple family members in the study? It is also not clear how the authors dealt with smoking by both ancestors. Did the analysis consider if both great-grandparents or grandparents were smokers? Based on other available literature which suggests an additive effect of parental smoking (more than just maternal or paternal alone) this is an important consideration and if it was not considered it should be clearly identified as a limitation of this study. It is not clear how the lack of change in lean mass is "a contrast to ensure that any effect of fat mass was not true of any other anthropometric measure". Finally there is a limited and vague discussion of why the associations in the great-grandchildren were not observed--this should be strengthened with some insights from other cohort studies or animal experiments.

Is the work clearly and accurately presented and does it cite the current literature?

Yes

If applicable, is the statistical analysis and its interpretation appropriate?

I cannot comment. A qualified statistician is required.

Are all the source data underlying the results available to ensure full reproducibility?

Yes

Is the study design appropriate and is the work technically sound?

Partly

Are the conclusions drawn adequately supported by the results?

Partly

Are sufficient details of methods and analysis provided to allow replication by others?

Yes

Reviewer Expertise:

Early life influences on metabolic health, parental smoking

I confirm that I have read this submission and believe that I have an appropriate level of expertise to confirm that it is of an acceptable scientific standard, however I have significant reservations, as outlined above.

# Response

**Published:** 2023-02-06

**Authors:** Yasmin Iles-Caven

**Affiliations:** University of Bristol, UK

Thank you very much for your constructive criticisms. These have been very useful in our rewriting of the paper. You will see that we have made the following changes in response to your review:

1. You were right to point out the confusion over numbers created by a reading of Table 1. We have revised this table and trust that it is now much clearer. In response to your query, there are no more than one grandchild/ great-grandchild per ancestor studied.
2. You questioned how we dealt with instances where both grandparents or great-grandparents smoked. We did not investigate this question due the small number of individuals with the required information. However, our results do suggest that only one member of each pair was particularly associated with fat mass in the (great) grandchildren. Examples pinpoint MGF but not MGM; PGF but not PGM (Table 2b), and PGFM but not PGFF (Table 4c). We have added this to the limitations.
3. We agree that it was unclear how the lack of change in lean mass is "a contrast to ensure that any effect of fat mass was not true of any other anthropometric measure". We have now clarified this in the first paragraph of the Discussion.
4. In response to your query about “why the associations in the great-grandchildren were not observed”, we have noted that these kinds of questions have arisen in rodent studies of smoke and nicotine exposure and remain unresolved, even though investigation of mechanism is relatively easier than in human studies.

# Response

**Published:** 2023-03-15

**Authors:** Yasmin Iles-Caven

**Affiliations:** University of Bristol, UK

Thank you very much for your constructive criticisms. These have been very useful in our rewriting of the paper. You will see that we have made the following changes in response to your review:

1. You were right to point out the confusion over numbers created by a reading of Table 1. We have revised this table and trust that it is now much clearer. In response to your query, there are no more than one grandchild/ great-grandchild per ancestor studied.
2. You questioned how we dealt with instances where both grandparents or great-grandparents smoked. We did not investigate this question due the small number of individuals with the required information. However, our results do suggest that only one member of each pair was particularly associated with fat mass in the (great) grandchildren. Examples pinpoint MGF but not MGM; PGF but not PGM (Table 2b), and PGFM but not PGFF (Table 4c). We have added this to the limitations.
3. We agree that it was unclear how the lack of change in lean mass is "a contrast to ensure that any effect of fat mass was not true of any other anthropometric measure". We have now clarified this in the first paragraph of the Discussion.
4. In response to your query about “why the associations in the great-grandchildren were not observed”, we have noted that these kinds of questions have arisen in rodent studies of smoke and nicotine exposure and remain unresolved, even though investigation of mechanism is relatively easier than in human studies.

# Peer review: Reviewer response for version 1

**DOI:** 10.21956/wellcomeopenres.19893.r52587

**Published:** 2022-10-18

**Authors:** Anthony P. Monaco

**Affiliations:** 1 Tufts University, Medford, MA, USA

This manuscript derives from a unique longitudinal study (ALSPAC) which has been utilized by this group in prior work to show associations between grand-paternal early onset nicotine smoking with fat mass in granddaughters and great granddaughters. In this manuscript they used fat mass, compared to lean mass, and took into account a number of possible associated factors with reduced cut-off for statistical significance.

 Although the approach seems sound, it needs to confirmed by a statistical expert. However, they do not define what 'lean mass' is and why it is an appropriate control. In addition, the data correlations and statistical analysis is presented in numerous tables. It is hard for the reader to discern on which of the many tables they should focus. May I suggest they consider moving some of tables to a supplement, and keep the most salient tables in the article. Is there any other way they can present the data which is more visual than tables full of numbers?

 Lastly, they make no effort to discuss possible mechanisms. Given the large volume of literature of substances of misuse in rodent models with offspring epigenetic effects, it seems like a paragraph in the discussion is warranted to point the reader to relevant animal models. It would also confirm that the transgenerational effects they are studying are not unique to humans.

Is the work clearly and accurately presented and does it cite the current literature?

Partly

If applicable, is the statistical analysis and its interpretation appropriate?

I cannot comment. A qualified statistician is required.

Are all the source data underlying the results available to ensure full reproducibility?

Yes

Is the study design appropriate and is the work technically sound?

Yes

Are the conclusions drawn adequately supported by the results?

Yes

Are sufficient details of methods and analysis provided to allow replication by others?

Yes

Reviewer Expertise:

Human genetics, epigenetics

I confirm that I have read this submission and believe that I have an appropriate level of expertise to confirm that it is of an acceptable scientific standard, however I have significant reservations, as outlined above.

# Response

**Published:** 2023-02-06

**Authors:** Yasmin Iles-Caven

**Affiliations:** University of Bristol, UK

Thank you for your helpful comments. In light of these we have made the following changes.

1. We have removed about half of the tables; They are now published in a Supplement.
2. We have described the lean mass measure and its possible importance in both the Introduction and the Discussion.
3. We have added more references in the Discussion in regard to the animal literature concerning transgenerational associations between maternal and paternal exposures to nicotine and to cigarette exposures. We have also speculated as to possible involvement of methylation changes. 

# Response

**Published:** 2023-03-15

**Authors:** Yasmin Iles-Caven

**Affiliations:** University of Bristol, UK

Thank you for your helpful comments. In light of these we have made the following changes.

1. We have removed about half of the tables; They are now published in a Supplement.
2. We have described the lean mass measure and its possible importance in both the Introduction and the Discussion.
3. We have added more references in the Discussion in regard to the animal literature concerning transgenerational associations between maternal and paternal exposures to nicotine and to cigarette exposures. We have also speculated as to possible involvement of methylation changes. 

# Peer review: Reviewer response for version 1

**DOI:** 10.21956/wellcomeopenres.19893.r51460

**Published:** 2022-07-26

**Authors:** Mathew Smith-Raska

**Affiliations:** 1 Division of Newborn Medicine, Department of Pediatrics, New York-Presbyterian Hospital, Weill Cornell Medicine, New York, USA

This is an important study using the ALSPAC dataset, examining the relationship between ancestral smoking and descendants’ fat mass. The authors have previously demonstrated that male smoking prior to puberty was associated with increased fat mass in their sons as well as granddaughters and great-granddaughters. In this study, they examine the relationship between ancestral smoking during adolescence with fat mass in subsequent generations at ages 17 and 24. As a control, they also examined lean mass. They report that adolescent smoking of the paternal grandfather is associated with increased fat mass of their grandchildren; there was a similar but weaker association with the maternal grandfather.

 This study benefits from the enormous ALSPAC dataset, which has potential to reveal many novel relationships between ancestral exposures and ancestral risk of disease. The authors very carefully and thoroughly consider multiple confounders that can potentially skew the results. 

 The authors do loosen the traditional p-value cutoff of < 0.05 for reasons explained in text, relating to being able to detect associations. Because I am not a statistician, I cannot comment on the validity of this approach. However, it should be considered that they are able to detect multiple associations using this loosened statistical stringency which supports the conclusion that these associations are true, however I am not qualified to state whether this is a valid approach.

 There are a lot of tables, and this manuscript may benefit from moving some to Supplemental Tables. I had some difficulty finding the important information among all of the tables. For example, under “Lean mass” on page 6: it implies that there are 23 of 24 positive associations in Table 2a, however there are only 14 of 25 positive associations in this table. Overall it was a bit difficult to find the most important information among all of the Tables. Examples that come to mind include Table 2b and Table 4b. I would recommend moving some of the Tables to a Supplemental Information section.

 The manuscript would also benefit from a better description of what “lean mass” is and how it differs from “fat mass,” including relevance to health/disease. Along similar lines, in the second-to-last paragraph it is stated that DXA measures of fat and lean mass are more accurate indicators than BMI; if mentioned there should be evidence or references to support this.

Minor point:

- Need to add a space between “ages” and “17” on Page 12 (currently reads as “ages17”)

Is the work clearly and accurately presented and does it cite the current literature?

Yes

If applicable, is the statistical analysis and its interpretation appropriate?

I cannot comment. A qualified statistician is required.

Are all the source data underlying the results available to ensure full reproducibility?

Partly

Is the study design appropriate and is the work technically sound?

Yes

Are the conclusions drawn adequately supported by the results?

Yes

Are sufficient details of methods and analysis provided to allow replication by others?

Yes

Reviewer Expertise:

Epigenetic Inheritance

I confirm that I have read this submission and believe that I have an appropriate level of expertise to confirm that it is of an acceptable scientific standard.

# Response

**Published:** 2023-02-06

**Authors:** Yasmin Iles-Caven

**Affiliations:** University of Bristol, UK

Thank you very much for your constructive criticisms. These have been very useful in our rewriting of the paper. You will see that we have made the following changes in response to your review:

1. We have taken your advice and moved 8 of the tables to a Supplementary file. Hopefully this will make the paper easier to read.
2. You were puzzled at the numbers of positive associations – this was meant to refer to the numbers of associations where the mean difference was +ve as opposed to -ve. This has been reworded to make it clearer that we were talking about an increase in mass, rather than a decrease.
3. You ask for a description of the differences between fat and lean mass, and why fat mass is a more relevant statistic than BMI. We have added a couple of sentences to the first paragraph of the Discussion and added relevant references.
4. We have corrected the typo.

# Response

**Published:** 2023-03-15

**Authors:** Yasmin Iles-Caven

**Affiliations:** University of Bristol, UK

Thank you very much for your constructive criticisms. These have been very useful in our rewriting of the paper. You will see that we have made the following changes in response to your review:

1. We have taken your advice and moved 8 of the tables to a Supplementary file. Hopefully this will make the paper easier to read.
2. You were puzzled at the numbers of positive associations – this was meant to refer to the numbers of associations where the mean difference was +ve as opposed to -ve. This has been reworded to make it clearer that we were talking about an increase in mass, rather than a decrease.
3. You ask for a description of the differences between fat and lean mass, and why fat mass is a more relevant statistic than BMI. We have added a couple of sentences to the first paragraph of the Discussion and added relevant references.
4. We have corrected the typo.
